# Decoding the medicinal aromatic characteristic of *Zhuyeqing* and gaining new insight into sotolon

**DOI:** 10.1016/j.fochx.2026.103630

**Published:** 2026-02-03

**Authors:** Lihua Wang, Yue Ma, Ying Han, Xing Zhang, Xiaojuan Gao, Wenshuo Li, Yanhong Bai, Fengxian Wang, Yan Xu, Qun Wu, Ke Tang

**Affiliations:** a Laboratory of Brewing Microbiology and Applied Enzymology, School of Biotechnology and Key Laboratory of Industrial Biotechnology of Ministry of Education, Jiangnan University, Wuxi 214122, China.; b Laboratory of Analytical, Quality Inspection Center, Key Laboratory of Plant Extraction and Health of Chinese Lujiu (Shanxi), Shanxi Xinghuacun Fenjiu Distillery Co., Ltd., Fenyang 032205, Shanxi, China.

**Keywords:** *Zhuyeqing*, Medicinal aromatic characteristic, Normal phase liquid chromatography, GC × GC-TOF/MS, GC-O, Key aroma compounds

## Abstract

*Zhuyeqing* is made from *Fenjiu* base liquor infused with extracts from 12 Chinese herbs. Its medicinal aroma is a unique feature of *Zhuyeqing.* However, this particular characteristic has not yet been thoroughly studied. Utilizing a normal phase liquid chromatography-sensomics-integrated strategy, we sequentially identified 110 potential odorants in the fraction with characteristic medicinal aroma via GC × GC-TOF MS, then these compounds were narrowed down to 25 sensorially active compounds through GC-O validation. Quantitative analysis identified 10 odorants with odor activity values (OAVs) ≥ 1. Omission experiments indicated that eugenol, sotolon, *trans*-isoeugenol, guaiacol, anisaldehyde, and acetoin were the key aroma compounds affecting the medicinal characteristic. Innovatively, we revealed the enantioselective distribution patterns of sotolon in *Zhuyeqing* and elucidated its dynamic evolution correlated with medicinal aroma attenuation. This study establishes the first molecular-level scientific framework for quality modulation of *Zhuyeqing*, bridging theoretical insights with practical applications.

## Highlights

• The medicinal aroma characteristics of *Zhuyeqing* were evaluated using sensomics.
• A total of 25 odorants were identified in the typical medicinal aroma fraction.
• The key medicinal aroma compounds of *Zhuyeqing* were confirmed.
• The composed ratios of (*R*)- and (*S*)-sotolon affected the medicinal aroma of *Zhuyeqing*.
• The (*R*)- and (*S*)-sotolon ratios in *Zhuyeqing* are not impacted by the bottled vintage.

## 1 Introduction

Herbs play a crucial role in the production of flavored alcoholic beverages (Liang et al., 2021). Internationally, flavored alcoholic beverages made from herbs and distilled spirits comprise a significant portion of the various types of flavored alcoholic beverages, such as *Zhizhonghe Wujiapi, Chinese JingJiu, and Zhuyeqing* (Ma et al., 2020; Sun et al., 2021). The addition of herbal extracts manipulates or enhances the flavor, taste, and health benefits of these beverages, catering to consumer preferences (Ma et al., 2020; Sun et al., 2021).

*Zhuyeqing*, a renowned traditional Chinese flavored spirit, is made from *Fenjiu* base liquor infused with extracts from 12 Chinese herbs (Wang, Han, et al., 2025). The strategic blending of 12 herbs imparts *Zhuyeqing* with a complex and distinctive aroma profile that defines its sensory identity. Its aroma profile features complementary notes of sweet, bitter almond, woody, medicinal, smoky, floral, fruity, acid, alcoholic (Wang, Han, et al., 2025. Each herbal from the 12 Chinese herbs contributes its medicinal aroma, such as cool, sweet, floral, woody, smoky, leafy, and herbal notes. Crucially, the medicinal aroma of *Zhuyeqing* differs fundamentally from isolated herbs or medicinal reagents. The unique medicinal aroma of *Zhuyeqing* arises from the synergistic interaction of the 12-herbal ensemble. Thus, the unique medicinal aroma of *Zhuyeqing* sets it apart from other herb-based beverages, which use different types of herbs, such as *Gins* (Buck et al., 2020), *Zhizhonghe Wujiapi* (Ma et al., 2020), *and Chinese JingJiu* (Sun et al., 2021), etc. Nonetheless, industrial evidence indicates progressive attenuation of its medicinal aroma during aging, highlighting a critical but unstudied molecular composition. This unresolved instability undermines the sensory characteristics of the beverage.

The aromas of alcoholic beverages are critical in determining the quality of the product and consumer preference (Tempere et al., 2019). Dunkel et al. (2014) established that the authentic aroma of each food can be sufficiently formed by only 3 to 40 key aroma compounds at natural concentrations, chosen from approximately 230 key food odorants out of around 10,000 food volatiles. Therefore, identifying the key aroma compounds from numerous candidates is essential but challenging (Wu et al., 2024). Research on the aromatic characteristics of Zhuyeqing is still limited. Recently, our investigations identified 883 volatile compounds in various types of Zhuyeqing (Wang, Zhang, et al., 2025) and successfully recreated the overall aroma profile (excluding the medicinal aroma) in dearomatized Zhuyeqing (Wang, Han, et al., 2025). While our prior study successfully confirmed that ethyl octanoate, ethyl cinnamate, *β*-damascenone, ethyl acetate, and eugenol are key odorants. However, the molecular composition of the typical medicinal aroma is still a mystery and needs to be investigated.

It is essential to have an efficient extraction and separation method for the analysis of aroma compounds in complex samples. The normal phase liquid chromatography (NPLC) effectively pre-separates the sample extracts, reducing matrix complexity and enhancing the identification accuracy (Zhao et al., 2021). To create mobile phases of varying polarities, reagents with different polarities are mixed in specific ratios. The resulting complex extracts are then pre-separated by elution on a silica gel column (Peppard, 1992). This approach isolates volatiles from diverse flavor matrices (Chen, Xu, & Qian, 2013; Fan et al., 2012; Zhao et al., 2021) and helps characterize signature aromas, such as the blackberry notes in *wine* (Falcao et al., 2012) and the oily odor in *Baijiu* (Wu et al., 2024).

Comprehensive two-dimensional gas chromatography-time-of-flight mass spectrometry (GC × GC-TOF MS) provides higher resolution and sensitivity for flavor analysis (Zou et al., 2023). This technique effectively resolves co-elution issues from the first dimension (Villière et al., 2012) and detects lower-abundance compounds *than conventional* gas chromatography–mass spectrometry (GC–MS). When combined with gas chromatography olfactometry (GC-O), GC × GC-TOF MS helps separate co-eluted compounds and identify those responsible for specific odors (Rochat et al., 2007; Villière et al., 2012).

The complex composition of Zhuyeqing causes overlapping chromatographic peaks. Some odorants, especially at very low concentrations, remain undetected. These issues make it difficult to identify compounds responsible for its medicinal aroma. Accordingly, we aim to: (1) explain the molecular composition of the typical medicinal aroma of *Zhuyeqing* using NPLC-GC × GC-TOF MS; (2) identify unique medicinal character odorants in *Zhuyeqing* via GC-O analysis; (3) quantify the odor-active compounds and calculate OAVs; (4) validate key odorant contributions through recombination/omission experiment.

## 2 Materials and methods

### 2.1 The samples

Six vintage *Zhuyeqing* samples between 0 and 25 (0, 3, 6, 11, 14, and 25 years) storage provided by Xinghuacun Fenjiu Distillery Co., Ltd. (hereinafter “Xinghuacun”, Shanxi, China) were involved in this study and all of them were produced based on traditional progress (Wang, Han, et al., 2025). Table S1 details bottled vintage *Zhuyeqing.* A panel of 10 certified national *Baijiu* tasting experts selected the representative medicinal aroma sample (*Zhuyeqing*-J0). All samples were stored in the dark at 25 °C before analysis.

### 2.2 Reagents and chemicals

HPLC grade reagents, including dichloromethane, absolute ethanol, pentane, and methanol, were supplied by Dikma Technologies Inc. (Beijing, China). Sodium chloride (NaCl), diethyl ether, and anhydrous sodium sulfate (Na_2_SO_4_) were bought from Sinopharm Chemical Reagent Co., Ltd. (Shanghai, China). Before use, dichloromethane, pentane, and diethyl ether were redistilled. All standard compounds and internal standards (ISs) (Table S2) were purchased from various suppliers and had a minimum purity of 95%.

### 2.3 Sensory analysis

This investigation was evaluated and authorized through the Jiangnan University IRB (JNU20230601IRB24). All procedures were strictly adhered to the Declaration of Helsinki and relevant laws and regulations, and no personally identifiable information about the participants was disclosed. Each participant provided informed consent before participating in this study and was free to withdraw at any time during the research period without consequences. The Jiangnan University IRB supervised this study fully and safeguarded the legitimate rights and interests of all participants.

A panel of 10 certified national *Baijiu* tasting experts (three males and seven females, aged from 27 to 40) from Xinghuacun was employed to evaluate the aroma profiles of the samples. They are responsible for the quality control of *Zhuyeqing*, familiar with the aroma characteristics of the 12 herbs of *Zhuyeqing*, and are able to identify different aroma characteristics precisely. After evaluating and discussing all bottled vintage *Zhuyeqing*, nine aroma descriptors, namely medicinal, sweety, alcoholic, nutty, woody, floral, fruity, smoky and acid were chosen (corresponding references are given in Table S3.) According to the tested samples, reference materials' aroma intensity has three levels referred to our previous work (Wu et al., 2024). The panelists judged the perception intensity of each attribute in the tested samples from 0 (none) to 6 (very strong). Each sample (~20 mL) labelled with three-digit codes was placed into glass cups at 25 °C.

### 2.4 Qualitative analysis of the medicinal aromatic characteristic of *Zhuyeqing*

#### 2.4.1 Aroma compound extraction methods

As previously reported method with minor changes (Wang, Han, et al., 2025), 100 mL (50 mL × 2) *Zhuyeqing*-J0 sample was diluted to 10% *v/v* ethanol using ultrapure water and was saturated using NaCl. The sample was then extracted with 50 mL mixture of organic solvents of pentane and diethyl ether (by volume ratio = 1:1) three times. All collected organic phases were combined and dried overnight using anhydrous Na_2_SO_4_. Then it was condensed to 2 mL using nitrogen under a slow-flowing stream, and kept at −20 °C before separation by NPLC.

Following Wu et al. (2024) described methods, NPLC was conducted to detect medicinal aroma compounds. The 2 mL concentrated sample was fractionated through a 30 cm glass column (1.5 cm i.d.) containing about 15 g of silica gel (63–200 μm, 70–230 mesh, Sigma-Aldrich, America Merck). The progress of the NPLC method was seen in Method S1. The eluent solvents, A (pentane), B (diethyl ether), and C (methanol), with the following volume ratios: F01: A (100), F02: A: B (95:5), F03: A: B (90:10), F04: A: B (80:20), F05: A: B (70:30), F06: A: B (50:50), F07: A: B (30:70), F08: A: B (20:80), F09: B (100), and F10: C (100). Ten fractions were collected and respectively concentrated to 1 mL before sensory and instrumental analysis by GC × GC-TOF MS and gas chromatography–mass spectrometry/olfactometry (GC–MS/O).

#### 2.4.2 Analysis with GC × GC-TOF MS.

In splitless mode, 1 μL sample was injected at 250 °C and the solvent delay time was 8 min. GC × GC-TOF MS with LECO Pegasus 4D (LECO, USA) and equipped with a gas chromatograph (Agilent 7890B) were used, and the analysis conditions were performed as previously reported (Wang et al., 2023) with slight changes. The columns (polar/moderately polar arrangement) were used for the GC × GC separation process. DB-FFAP (60 m × 0.25 mm i.d., 0.25 μm, Agilent, USA) as the first column and Rxi-17 Sil MS Cap. column (1.5 m × 0.25 mm i.d., 0.25 μm, Restek, USA) as the second column, respectively. The analysis time was total 45.75 min and the program oven temperature of the first column was set as the DB-FFAP in Method S2. Besides, the second oven temperature was maintained set a higher 5 °C than that of the first oven. The modulator between two columns was installed as a quad-jet and a dual-stage thermal. Set the hot pulse time at 1.2 s and the modulation period at 6 s. Finally, the transmission line was at 250 °C, and the temperature of the ionization source was at 230 °C for the TOF MS system, respectively.

#### 2.4.3 Analysis with GC–MS/O

A gas chromatograph (Agilent 7890 A) coupled with a detector of mass selective (Agilent 5977B) and an ODP 2 olfactometric system (Gerstel, Germany) (GC–MS/O) was used. Compound separation and GC-O analysis (GC-O passed the ethical review and the licensed number was JNU20230601IRB24) were performed with a 60 m column of DB-FFAP (0.25 mm i.d., 0.25 μm, Agilent, USA) and a 30 m column of DB-5 (0.25 mm i.d., 0.25 μm, Agilent, USA). At 250 °C, 1 μL sample was injected with a 5 min solvent delay and in splitless mode. As in our previous paper (Wang, Han, et al., 2025), the parameter setting for GC–MS/O was seen in Method S2. Osme technology was used to perform GC-O. Three trained assessors (two females and one male) sniffed the sample, and the odor could be regarded as existing after at least two recordings. The qualitative method involves comparing the retention index (RI), mass spectrometry, and aroma descriptions with established standards in DB-FFAP and DB-5 columns.

### 2.5 Quantitation of odorants

The standard curves were plotted using peak area ratios (test chemical/internal standard) on the vertical axis and concentration ratios on the horizontal axis (Table S4). To ensure accuracy, triplicate measurements were performed for all samples, with all curves demonstrating linearity (R^2^ ≥ 0.99). Based on the sensitivity of compounds, two quantification methods, as follows, were used.

#### 2.5.1 Quantification with liquid-liquid extraction (LLE) and GC × GC-TOF MS

Seventeen aromas were quantified using the LLE-GC × GC-TOF MS method. The samples (50 mL) added with 50 μL of mixed ISs were pretreated and extracted with 50 mL CH_2_Cl_2_ (repeated 3 times) as described above. The mixed ISs contain ethyl octanoate-d15 (IS1, 500.00 mg/L), 1-butanol-d10 (IS2, 500.00 mg/L), l-menthol (IS3, 100.00 mg/L), 2-methoxyphenol-d3 (IS4, 100.00 mg/L), benzyl alcohol-d7 (IS5, 100.00 mg/L), and sotolon-^13^C2 (IS6, 100.00 mg/L) with final concentrations, respectively. One microliter (1 μL) sample was injected with splitless mode at 250 °C and the solvent delay time was 8 min. The GC × GC-TOF MS analysis was conducted as described in section 2.4.2. without change.

#### 2.5.2 Quantification with liquid-liquid microextraction (LLME) and GC–MS

As our previously reported (Wang, Han, et al., 2025), six compounds were quantified with LLME-GC–MS. Samples (8 mL each) were diluted to 10% ethanol (*v*/v) with saturated NaCl solution, followed by the addition of 50 μL mixed ISs: 2-methoxyphenol-d3 (IS4, 100.00 mg/L) and benzyl alcohol-d7 (IS6, 100.00 mg/L). LLME was performed with CH_2_Cl_2_ (5 mL per cycle, triplicate) under vortex mixing (800 rpm, 5 min), after which the extract was concentrated to 1 mL. GC–MS analysis was conducted under the conditions outlined for the DB-FFAP column in Method S2.

### 2.6 Determination of the odor thresholds

The odor thresholds in 45% vol ethanol/water of sotolon, piperonyl acetone, 3-methyl-2-cyclohexen-1-one, *β*-isophorone, (*R*)*-*sotolon, and (*S*)*-*sotolon were determined to calculate OAVs using the ten-sample test (TST) method as our previously stated procedure (Wang, Han, et al., 2025).

### 2.7 Aroma recombination and omission tests

As our previously stated procedure (Wang, Han, et al., 2025), the dearomatized *Zhuyeqing* was prepared (seen Method S4). Two group reconstitution experiments in 45% vol dearomatized *Zhuyeqing* were evaluated against the typical original *Zhuyeqing* (*Zhuyeqing-*J0) by 10 trained panelists to validate the aroma contributions in medicinal character. Group 1 contains the 22 odorants (OAVs ≥1) found in the overall profile research (Table S5, as detailed in Table 3 in our earlier paper (Wang, Han, et al., 2025)). Group 2 contains 28 odorants (OAVs ≥1). Compared to group 1, another 6 newly identified medicinal compounds with OAV > 1 in F10 (Table 4) were added. All these compounds were added based on their actual concentrations in the typical original *Zhuyeqing*. Two group reconstitution models and one typical original *Zhuyeqing* sample were assessed by 10 panelists, who rated the intensity of seven aroma attributes on a scale from 0 (none) - 6 (strong).

Eleven tests of aroma omission (Table 5) were performed in dearomatized *Zhuyeqing* and compared against the complete recombination model in dearomatized *Zhuyeqing* to determine the importance of specific compounds by a published triangle test methodology (Wang, Han, et al., 2025). For evaluation, three samples (~20 mL), one omission and two complete models, were presented simultaneously in glasses. Ten assessors identified the sample exhibiting the most pronounced sensory difference. Testing was conducted in triplicate, with results subjected to statistical analysis.

### 2.8 Distribution of the sotolon enantiomers in *Zhuyeqing*

#### 2.8.1 HPLC separation and GC–MS conditions for (*R*)- and (*S*)-sotolon

The (*R*)- and (*S*)-sotolon enantiomers were isolated and purified by Daicel Chiral Technologies Co., Ltd. (Shanghai, China) using their HPLC (Shimadzu LC-20AD) with Chiralpak IH column (IH00CE-CX015, 25 cm × 0.46 cm). The mobile phase was a mixture of ethanol/n-hexane 20:80 (*v*/v) with a flow rate of 1.0 mL/min at a constant temperature of 35 °C and detection was performed at 250 nm.

GC–MS (7890 A-5977B, Agilent, USA) was used to identify the retention times of (*R*)- and (*S*)-sotolon, and analyze them in *Zhuyeqing* extracts. The separation columns were DB-FFAP (2 m × 0.25 mm i.d., 0.25 μm, Agilent, USA) connected with HP-Chiral 20*β* (30 m × 0.25 mm i.d., 0.25 μm, Agilent, USA). Oven parameters refer to the published method with minor changes (Pons et al., 2008), starts at 50 °C and hold for 0.1 min, with 1 °C/min raised to 110, hold for 20 min, then with 3 °C/min raised to 210 °C and hold for 20 min. 1 μL sample was injected in splitless mode at 250 °C with a 5 min solvent delay.

#### 2.8.2 Sotolon enantiomers extraction from *Zhuyeqing* samples

The LLME-GC–MS method was used to identify the distribution and explore the origins of sotolon enantiomers in *Zhuyeqing*. Each sample (20 mL) was pretreated and extracted with 20 mL CH_2_Cl_2_ (repeated in triplicate) and concentrated to 1 mL as described above, then 1 μL sample was injected.

### 2.9 Statistical analysis

The SPSS software for Windows (version 26.0, Chicago, IL, USA) was utilized for the statistical analyses. The data from aroma profiling and quantitative analysis were evaluated using analysis of variance (ANOVA) in SPSS.

## 3 Results and discussion

### 3.1 Sensory analysis of bottled vintage *Zhuyeqing* samples

**Fig. 1:**
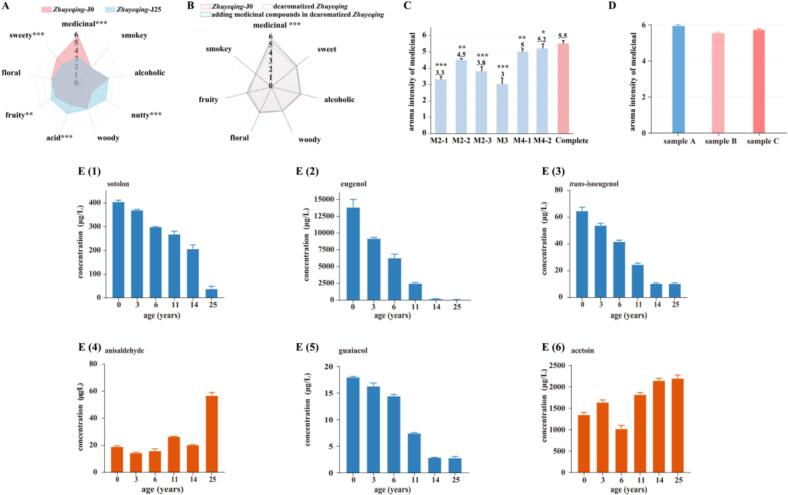
Aroma profiles of *Zhuyeqing-*J0 and *Zhuyeqing-*J25 samples (A); Aroma profile analyses of *Zhuyeqing-*J0 and the complete aroma reconstitution model in dearomatized *Zhuyeqing* (B); Intensity score of medicinal aroma in omission groups (C); Intensity score of medicinal aroma in *Zhuyeqing* and the complete aroma reconstitution model in dearomatized *Zhuyeqing* sample: sample A (*Zhuyeqing-*J0), sample B (with real concentrations of the commercial racemic sotolon in *Zhuyeqing-*J0), sample C (with real concentrations of (*R*)-sotolon and (*S*)-sotolon in *Zhuyeqing-*J0) (D). The scores of selected descriptors were averages evaluated by 10 panelists. *, ** and *** indicate significance at *p* < 0.05, *p* < 0.01 and *p* < 0.001, respectively. (E) Concentrations of medicinal aroma compounds in bottled vintage *Zhuyeqing* samples.

Combined with our previous research on typical *Zhuyeqing* (Wang, Han, et al., 2025), the nine agreed-upon aroma descriptors of alcoholic, nutty, floral, woody, smoky, fruity, acid, sweety, and medicinal are the typical profiles of the *Zhuyeqing* (Fig. 1A). The sensory analysis results showed no significant differences among alcoholic, floral, woody, and smoky aromas. However, the intensities of the sweety and medicinal aromas in *Zhuyeqing*-J0 were significantly higher than those in *Zhuyeqing*-J25. Conversely, the intensities of fruity, acid, and nutty aromas in *Zhuyeqing*-J0 were significantly lower than those in *Zhuyeqing*-J25. Our earlier research indicated that the intensity of the reconstituted medicinal aroma significantly differed from that of the typical original *Zhuyeqing* (Wang, Han, et al., 2025). Sensory analysis of bottled vintage *Zhuyeqing* demonstrated that the intensities of medicinal aromas significantly diminish during aging. And our previous research directly using LLE and GC–MS/O did not identify a single substance responsible for the medicinal aroma. Therefore, this typical characteristic of *Zhuyeqing* was further investigated using the method of LLE-NPLC combined with GC × GC-TOF/MS and GC–MS/O.

### 3.2 Identification of medicinal aroma compounds in *Zhuyeqing* with NPLC

To determine the aroma components corresponding to the medicinal aroma attributes of *Zhuyeqing*, its LLE extraction was fractionated and separated by NPLC, and a total of 10 fractions were obtained. Sensory analysis revealed that the individual fractions exhibited different aroma characteristics (Table 1), among which F10 had the most typical medicinal aroma characteristics of *Zhuyeqing*. Thus, the aroma components with medicinal attributes could be locked in more quickly by sniffing F10.

**Table 1:** Aroma description of fractions obtained by NPLC.

Fraction	Aroma descriptions a	Aroma intensity score b
F01	sweety, floral	3.3
F02	floral, fruity, cool, sweety, *Danggui*	5.4
F03	rancid, leather, *Muxiang*, *Tangxiang*	3.2
F04	animal, petrol, smoky, *Zhuye*	1.5
F05	sweety, floral, likely *Sharen*	1.8
F06	rancid, sweety, likely *Juhua*	5.3
F07	vinegar, rancid, sweety, likely *Zhizi*	5.5
F08	petrol, medicinal	1.5
F09	nutty, cool, medicinal, little smoky	4.5
F10	medicinal, sweety, little smoky	5.8

a The aroma descriptions were discussed by 10 panelists.

b The aroma intensity from “0–2” scores indicated “none” to “low”, “2–4” scores indicated “medium”, and “4–6” scores indicated high, respectively. The aroma intensity of those fractions were averages evaluated by 10 panelists.

Applying GC × GC-TOF/MS, a total of 110 substances with aroma characteristics in F10 were identified through the search of three flavor libraries, Flavor DB (https://cosylab.iiitd.edu.in/flavordb/), Flavornet Home (http://www.flavornet.org), and OlfactionBase (https://olfab.iiita.ac.in/olfactionbase/) (Table 2).

**Table 2:** 110 aroma compounds in F10 resolved by GC × GC-TOF/MS and their aroma characteristics.

No.	CAS	Name	Similarity	LRIcal ^*a*^	LRIlit ^*b*^	Identification ^*c*^	Aroma descriptions ^*d*^
1	600–14–6	2,3-pentanedione	774	1065	1062	RI, MS	sweet, butter, creamy, caramel, nutty
2	110–43-0	2-heptanone	932	1192	1213	RI, MS, STD	soap
3	106–70-7	methyl hexanoate	797	1195	1221	RI, MS, STD	fruit, fresh, sweet
4	3777-69-3	2-pentylfuran	730	1230	1241	RI, MS, STD	green bean, butter
5	505–57-7	(*E*)-2-hexenal	733	1233	1216	RI, MS, STD	apple, green
6	123–66-0	ethyl hexanoate	756	1239	1267	RI, MS, STD	sweet, fruity, pineapple, green, banana
7	106–68-3	3-octanone	874	1264	1252	RI, MS, STD	fresh, herbal, lavender, sweet, mushroom
8	100–42-5	styrene	953	1270	1300	RI, MS, STD	sweet, balsam, floral, plastic
9	111–13-7	2-octanone	930	1293	1309	RI, MS, STD	herb, butter, resin
10	106–73-0	methyl heptanoate	779	1296	1327	RI, MS, STD	sweet, fruity, and green
11	124–13-0	octanal	913	1299	1319	RI, MS, STD	fat, soap, lemon, green
12	513–86-0	acetoin	776	1320	1287	RI, MS, STD	sweet, buttery, creamy, dairy, milky
13	2408-37-9	2,2,6-trimethylcyclohexanone	931	1329	1333	RI, MS	pungent, honey, cistus
14	585–25-1	2,3-octanedione	748	1332	1376	RI, MS, STD	herbal, fatty
15	18829–55-5	(*E*)-2-heptenal	855	1337	1339	RI, MS, STD	soap, fat, almond
16	3188-00-9	2-methyltetrahydrofuran-3-one	798	1340	1242	RI, MS	sweet, solvent, bread, buttery, nutty
17	110–93-0	methylheptenone	813	1346	1342	RI, MS	pepper, mushroom, rubber
18	97–64-3	ethyl lactate	879	1348	1309	RI, MS, STD	fruit
19	111–11-5	methyl octanoate	935	1393	1437	RI, MS, STD	green, sweet, aldehydic, vegetable, herbal
20	821–55-6	2-nonanone	902	1396	1367	RI, MS, STD	fresh, sweet, green, weedy, earthy, herbal
21	124–19-6	nonanal	919	1401	1422	RI, MS, STD	fat, citrus, green
22	471–01-2	*β*-isophorone	706	1420	1416	RI, MS, STD	woody, camphor, musty, tobacco, leather
23	106–32–1	ethyl octanoate	792	1434	1477	RI, MS, STD	fruity, sweet, apricot, banana, brandy, pear
24	2548-87-0	(*E*)-2-octenal	911	1438	1433	RI, MS, STD	green, nut, fat
25	498–60–2	3-furaldehyde	920	1440	1438	RI, MS, STD	sulphurous
26	1195-32-0	1-methyl-4-isopropenylbenzene	902	1445	1428	RI, MS	citrus, pine
27	591–12-8	5-methyl-2(3*H*)-furanone	823	1449	1429	RI, MS, STD	sweet, creamy, coconut, vanilla
28	64–19-7	acetic acid	963	1453	1465	RI, MS, STD	sharp, pungent, sour, vinegar
29	930–68-7	2-cyclohexen-1-one	857	1454	1462	RI, MS, STD	savory, green, pesticide, roasted
30	98–01-1	furfural	957	1478	1486	RI, MS, STD	sweet, woody, almond, fragrant, baked bread
31	104–76-7	2-ethylhexanol	850	1485	1484	RI, MS, STD	citrus, fresh, floral, oily, sweet
32	271–89-6	benzofuran	913	1519	1486	RI, MS, STD	styrene, aromatic
33	109–97-7	pyrrole	882	1524	1521	RI, MS	sweet, warm, nutty, ethereal
34	100–52-7	benzaldehyde	849	1537	1568	RI, MS, STD	strong, sharp, sweet, almond
35	24347–58-8	(*R, R*)-2,3-butanediol	960	1538	1580	RI, MS, STD	fruity, creamy, buttery
36	79–09-4	propanoic acid	843	1540	1486	RI, MS, STD	cheesy, vinegar
37	10348–47-7	ethyl 2-hydroxy-4-methylpentanoate	903	1542	1547	RI, MS	fresh, blackberry
38	79–31-2	2-methylpropanoic acid	928	1566	1574	RI, MS, STD	acidic, sour, cheese, dairy, buttery, rancid
39	19132–06-0	(*S, S*)-2,3-butanediol	972	1578	1580	RI, MS, STD	fruity, creamy, buttery
40	110–42-9	methyl decanoate	882	1598	1613	RI, MS, STD	wine
41	106–65-0	dimethyl butanedioate	842	1603	1526	RI, MS, STD	winey, sweet, green, fruity, floral
42	112–12-9	2-undecanone	889	1604	1592	RI, MS, STD	orange, fresh, green
43	78–59-1	isophorone	899	1610	1621	RI, MS, STD	woody, camphor, musty, tobacco, leather
44	1193-18-6	3-methyl-2-cyclohexen-1-one	935	1618	1579	RI, MS, STD	caramel, phenolic, almond, sweet, medicinal
45	108–29-2	*γ*-valerolactone	905	1636	1589	RI, MS, STD	herbal, sweet, tobacco, cocoa
46	107–92-6	butanoic acid	834	1637	1628	RI, MS, STD	sharp acetic, cheese, butter, fruit
47	93–58-3	methyl benzoate	894	1642	1637	RI, MS, STD	prune, lettuce, herb, sweet
48	96–48-0	4-hydroxybutanoic acid lactone	873	1658	1643	RI, MS, STD	creamy, oily, fatty, caramel
49	3913-81-3	(*E*)-2-decenal	815	1658	1641	RI, MS, STD	tallow, waxy, fatty, earthy, coriander, mushroom
50	122–78-1	phenylacetaldehyde	797	1664	1650	RI, MS, STD	hawthorne, honey, sweet
51	98–86-2	acetophenone	706	1672	1655	RI, MS, STD	medicinal, must, sweet, almond, pungent
52	109–52–4	pentanoic acid	860	1678	1733	RI, MS, STD	sweat, acidic, dairy-like with milky, cheese
53	93–89-0	ethyl benzoate	962	1683	1706	RI, MS, STD	fruity, dry, musty, sweet, wintergreen
54	123–25-1	diethyl butanedioate	913	1687	1694	RI, MS, STD	mild, fruity, cooked, apple, ylang
55	112–17-4	decyl acetate	844	1687	1680	RI, MS, STD	soapy, pineapple, orange, clean, citrus
56	1731-86-8	methyl undecanoate	902	1705	1703	RI, MS, STD	waxy, rose, fruity, fatty
57	1125-21-9	ketoisophorone	933	1707	1677	RI, MS, STD	citrus, floral, musty, tea like with green
58	22104–80-9	2-decen-1-ol	786	1719	1794	RI, MS, STD	waxy, citrus
59	112–54-9	dodecanal	923	1719	1709	RI, MS, STD	soapy, citrus, green, floral
60	695–06-7	*γ*-hexalactone	871	1725	1703	RI, MS, STD	sweet, creamy, vanilla, lactonic, powdery
61	4748-78-1	4-ethyl benzaldehyde	870	1728	1730	RI, MS, STD	sweet
62	93–55-0	propiophenone	843	1743	1734	RI, MS, STD	hawthorn, clove
63	89–81-6	piperitone	747	1746	1730	RI, MS, STD	herbal, minty, camphor, pepper
64	99–75-2	methyl 4-methylbenzoate	919	1766	1725	RI, MS, STD	woody, camphor
65	15764–16-6	2,4-dimethylbenzaldehyde	839	1769	1710	RI, MS, STD	cherry, almond, vanilla
66	497–23-4	2(5*H*)-furanone	865	1783	1787	RI, MS, STD	buttery
67	2407-43-4	5-ethyl-2(5*H*)-furanone	852	1784	1734	RI, MS, STD	spices
68	111–82-0	methyl dodecanoate	906	1809	1814	RI, MS, STD	fat, waxy, soapy, creamy, coconut, mushroom
69	105–21-5	*γ*-heptalactone	828	1827	1796	RI, MS, STD	nut, fat, fruit
70	118–61-6	ethyl 2-hydroxybenzoate	863	1831	1791	RI, MS, STD	balsamic, cool, herbal, minty, spices, sweet
71	542–28-9	*δ*-valerolactone	779	1835	1780	RI, MS, STD	fatty, fruity, lactonic
72	104–46-1	anethole	826	1846	1834	RI, MS, STD	sweet, anise, licorice, medicinal
73	58461–27-1	(+/−)-lavandulol	719	1868	nd	MS	delicate, farnesol-like, floral, herbal, mimosa
74	90–05-1	guaiacol	900	1879	1842	RI, MS, STD	phenolic, smoke, spice, vanilla, woody
75	57194–69-1	(*Z*)-3-phenylacrylaldehyde	818	1890	1888	RI, MS, STD	spices, cinnamyl
76	104–55-2	cinnamaldehyde	760	1890	1978	RI, MS, STD	sweet, spicy, aldehydic, cinnamyl
77	937–30-4	4′-ethylacetophenone	910	1890	1867	RI, MS	floral, hawthorn
78	100–51-6	benzyl alcohol	777	1894	1895	RI, MS, STD	sweet, floral, cherry, almond
79	60–12-8	phenylethanol	897	1929	1931	RI, MS, STD	honey, spice, rose, clove
80	104–50-7	*γ*-octalactone	910	1943	1924	RI, MS, STD	sweet, coconut, creamy
81	111–14-8	heptanoic acid	734	1961	1960	RI, MS, STD	waxy, cheesy, fruity, dirty and fatty
82	93–51-6	creosol	899	1979	1956	RI, MS, STD	spicy, clove, vanilla, medicinal, leathery, smoky
83	95–16-9	benzothiazole	867	1988	1958	RI, MS, STD	sulfury, cooked, nutty, coffee
84	95–48-7	2-methylphenol	722	2019	1996	RI, MS, STD	phenol
85	108–95-2	phenol	795	2024	2030	RI, MS, STD	phenolic, plastic, rubber
86	93–15-2	methyl eugenol	950	2030	2042	RI, MS, STD	vanilla, spicy, clove, sweet, fresh, cinnamon
87	123–11-5	anisaldehyde	948	2063	2045	RI, MS, STD	mint, sweet
88	124–07-2	octanoic acid	734	2069	2070	RI, MS, STD	fatty, waxy, rancid, oily, vegetable, cheesy
89	106–44-5	*p*-cresol	910	2102	2089	RI, MS, STD	medicine, phenol, smoke
90	104–45-0	1-methoxy-4-propylbenzene	708	2102	1606	RI, MS, STD	anisic, fennel, herbal, licorice
91	108–39-4	3-methylphenol	883	2114	2077	RI, MS, STD	fecal, plastic
92	41114–00-5	ethyl pentadecanoate	914	2162	2140	RI, MS, STD	sweet, honey
93	112–05-0	nonanoic acid	856	2173	2169	RI, MS, STD	green, fat
94	97–53-0	eugenol	968	2196	2144	RI, MS, STD	sweet, spicy, clove, woody
95	620–17-7	3-ethylphenol	848	2202	2171	RI, MS, STD	earthy/ musty, musty, musty/moldy
96	7786-61-0	4-vinylguaiacol	791	2226	2175	RI, MS, STD	clove, curry, smoky, bacon
97	112–39-0	methyl hexadecanoate	928	2227	2233	RI, MS, STD	oily, waxy, fatty, orris
98	28664–35-9	sotolon	788	2231	2216	RI, MS, STD	herb and smoky
99	334–48-5	decanoic acid	806	2279	2279	RI, MS, STD	rancid, fat
100	120–57-0	piperonal	810	2285	2244	RI, MS	vanilla, herbal, heliotrope, floral, cinnamyl
101	91–10-1	2,6-dimethoxyphenol	900	2294	2273	RI, MS, odor	bacon, woody, sweet (medicinal), smoky
102	36653–82–4	1-hexadecanol	856	2381	2400	RI, MS, STD	wax, floral
103	5932-68-3	*trans*-isoeugenol	828	2386	2332	RI, MS, STD	sweet, spicy, clove, woody, allspice, carnation
104	65–85-0	benzoic acid	907	2465	2446	RI, MS, STD	balsamic, urine, faint
105	120–72-9	indole	705	2493	2450	RI, MS, STD	animal, fecal with earthy, perfumey, phenolic
106	67–47-0	5-hydroxymethylfurfural	760	2539	2528	RI, MS, STD	herbal, hay, tobacco
107	55418–52-5	piperonyl acetone	762	2577	nd	MS, STD	cotton candy, heliotrope, powdery, sweet
108	121–33-5	vanillin	877	2618	2571	RI, MS, STD	vanilla, sweet, creamy, spicy, phenolic, milky
109	498–02–2	acetovanillone	812	2685	2640	RI, MS	almond, burnt, coffee, earthy/musty, vanilla
110	57–10-3	n-hexadecanoic acid	836	2914	2913	RI, MS, STD	waxy, creamy fatty

LRIcal ^a^: calculated linear retention indices. LRIlit ^b^: literature linear retention indices obtained from the NIST library (https://webbook.nist.gov/chemistry/), “nd” = not determined. Identification ^c^: identification based on retention indices (RI), mass spectra (MS), and retention times of authentic standards (STD). odor ^*d*^: searching from two flavor libraries, Flavor DB (https://cosylab.iiitd.edu.in/flavordb/) and Flavornet Home (http://www.flavornet.org).

F10 was used to search medicinal contribution aromas by GC–MS/O, and 25 odorants were confirmed (Table 3). Combining the results of GC × GC-TOF/MS with GC–MS/O, a total of 23 aromas were finally identified, but two odorants still could not be identified. Specifically, the following odorants were not found in the overall profile research using the GC-O with LLE concentration of *Zhuyeqing* (Wang, Han, et al., 2025), such as sotolon, isophorone, *β*-isophorone, 3-methyl-2-cyclohexen-1-one, benzoic acid methyl ester, acetophenone, piperitone, anethole, anisaldehyde, 2,6-dimethoxyphenol, and piperonyl acetone. Sotolon contributes to diverse aroma profiles in different samples. In alcoholic beverages, it could contribute a caramel-like character (Chen, Wang, & Xu, 2013; Wang et al., 2023), curry, walnut, or rancid notes (Pons et al., 2008), *woody nuances* (Janáčová et al., 2008). However, in plant-based samples, sotolon displays lovage-like and seasoning-like qualities (Marcinkowska et al., 2021). Acetophenone was found in *Jiangxiangxing* (Wang et al., 2020) and *Fengxiangxing* (Jia et al., 2022) *Baijiu* with medicinal, almond, and pungent aroma. Most recently, piperitone, a monoterpene ketone with pepper and minty aroma, was first identified and quantified in *Baijiu* by Wang, Gao, et al. (2024). Anethole and anisaldehyde with anise odor were found to greatly impact the overall aroma of *Wujiapi* medicinal liquor (Ma et al., 2020; Ma et al., 2022). 2,6-dimethoxyphenol with the woody and medicinal smell was found in *JingJiu* (Sun et al., 2021).

**Table 3:** 25 aroma compounds in F10 with the medicinal character of *Zhuyeqing* by GC–MS/O.

No.	CAS	Compounds	RI ^*a*^		RT (s)	Aroma description ^*b*^	Identification ^*c*^	Intensity values in FFAP
			FFAP	DB-5				
1	513–86-0	acetoin	1292	731	972, 1.50	sweet, creamy, milky	RI, MS, STD, odor	4.7
2	78–59-1	isophorone	1415	1116	1506, 2.01	woody, leather	RI, MS, STD, odor	3.8
3	471–01-2	*β*-isophorone	1420	1039	1200, 2.15	floral and hay	RI, MS, STD, odor	4.2
4	24,347–58-8	(*R, R*)-2,3-butanediol	1539	nd	1404, 1.37	fruity	RI, MS, STD, odor	3.5
5	100–52-7	benzaldehyde	1540	961	1404, 1.75	sweet, almond	RI, MS, STD, odor	4.0
6	19,132–06-0	(*S, S*)-2,3-butanediol	1578	nd	1464, 1.35	fruity, onion	RI, MS, STD, odor	3.0
7	1193-18-6	3-methyl-2-cyclohexen-1-one	1613	nd	1518, 1.89	phenolic, almond, medicinal	RI, MS, STD, odor	3.2
8	93–58-3	methyl benzoate	1634	1103	1548, 1.92	herb, sweet	RI, MS, STD, odor	4.8
9	98–86-2	acetophenone	1665	1051	1596, 1.91	medicinal, sweet, almond	RI, MS, STD, odor	5.0
10	89–81-6	piperitone	1746	nd	1716, 2.09	herbal, minty, pepper	RI, MS, STD, odor	4.8
11	104–46-1	anethole	1842	1832	1848, 1.74	sweet, anise, medicinal	RI, MS, STD, odor	5.4
12	90–05-1	guaiacol	1879	1086	1884, 1.45	phenolic, smoke, spice, woody	RI, MS, STD, odor	5.0
13	100–51-6	benzyl alcohol	1886	1034	1902, 1.39	sweet, floral, cherry	RI, MS, STD, odor	5.4
14	60–12-8	phenylethanol	1920	1110	1938, 1.43	honey, spice, rose	RI, MS, STD, odor	5.2
15	108–95-2	phenol	2024	986	2028, 1.31	phenolic, plastic	RI, MS, STD, odor	4.0
16	123–11-5	anisaldehyde	2056	1248	2058, 1.50	woodruff-like, anise-like	RI, MS, STD, odor	5.0
17	nd	unknown 1	2138	nd	nd	medicinal, smoke	odor	4.8
18	97–53-0	eugenol	2185	1353	2154 1.49	sweet, spicy, clove, woody	RI, MS, STD, odor	5.6
19	7786-61-0	4-vinylguaiacol	2219	1308	2178,1.48	clove, smoky, bacon	RI, MS, STD, odor	5.5
20	28,664–35-9	sotolon	2231	1090	2184, 1.34	herb and smoky	RI, MS, STD, odor	5.8
21	91–10-1	2,6-dimethoxyphenol	2294	nd	2232, 1.54	woody, medicinal, smoky	RI, MS, STD, odor	3.6
22	5932-68-3	*trans*-isoeugenol	2386	1445	2298, 1.63	sweet, spicy, clove, woody	RI, MS, STD, odor	5.0
23	nd	unknown 2	2397	nd	nd	smoke, clove	odor	4.2
24	55,418–52-5	piperonyl acetone	2571	1588	2466, 2.01	cotton candy, heliotrope, sweet	RI, MS, STD, odor	4.0
25	121–33-5	vanillin	2618	1391	2514, 1.69	vanilla, sweet, spicy	RI, MS, STD, odor	3.2

RI ^***a***^ = retention index on different stationary phases; nd = not determined. **Odor description**^b^: search from three flavor libraries: Flavor DB (https://cosylab.iiitd.edu.in/flavordb/), Flavornet Home (http://www.flavornet.org), and OlfactionBase (https://olfab.iiita.ac.in/olfactionbase/). Identification ^c^: based on retention indices (RI), mass spectra (MS), and authentic standards (STD), or odor description.

### 3.3 Quantification and OAV analysis of aromatic compounds

Twenty-three compounds were quantified with LLME-GC/MS and LLE-GC × GC-TOF MS quantification methods (Table 4) on the medicinal aroma research of *Zhuyeqing*. Among them, sotolon, eugenol, anisaldehyde, acetoin, (*R, R*)-2,3-butanediol, (*S, S*)-2,3-butanediol, guaiacol, isophorone, phenol, and *trans*-isoeugenol had OAVs ≥1.00, the other compounds with OAVs <1.00.

**Table 4:** Concentrations, odor thresholds, and OAVs in F10 with the medicinal character of *Zhuyeqing*).

Compounds	Odor threshold (μg/L)	Concentrations (μg/L)	OAV
sotolon	10.70 a	602.40 ± 15.64	56
eugenol	470.00 (Fan et al., 2015)	13,617.04 ± 388.13	29
anisaldehyde	21.00 (Ma et al., 2020)	234.00 ± 0.67	11
acetoin	259.00 (Fan et al., 2015)	1650.42 ± 0.32	6
(*R,R*)-2,3-butanediol	95.10 (Gemert, 2011)	537.50 ± 0.25	6
(*S,S*)-2,3-butanediol	668.00 (Gemert, 2011)	2152.50 ± 0.32	3
guaiacol	9.50 (Guo et al., 2022)	23.70 ± 0.39	3
isophorone	11.00 (Guo et al., 2022)	22.07 ± 0.06	2
phenol	30.00 (Guo et al., 2022)	59.43 ± 4.05	2
*trans*-isoeugenol	22.54 (Fan & Xu, 2011)	35.53 ± 1.62	2
3-methyl-2-cyclohexen-1-one	1000 a	814.80 ± 1.91	0.81
vanillin	438.52 (Fan & Xu, 2011)	106.70 ± 0.03	0.24
4-vinylguaiacol	209.30 (Fan & Xu, 2011)	47.27 ± 0.19	0.23
piperonyl acetone	50 a	9.80 ± 0.15	0.2
benzaldehyde	515.00 (Zhang et al., 2020)	56.25 ± 7.01	0.11
2-phenylethanol	40,000.00 (L. Wang et al., 2024)	2368.18 ± 40.49	0.06
2,6-dimethoxyphenol	1384.25 (Sun et al., 2021)	69.17 ± 0.01	0.05
*β*-isophorone	30 a	1.50 ± 0.15	0.02
acetophenone	65 (Gemert, 2011)	0.95 ± 0.22	0.01
anethole	36.00 (Ma et al., 2020)	0.40 ± 0.00	0.01
benzyl alcohol	40,900.00 (Fan et al., 2015)	61.06 ± 6.62	<0.01
piperitone	2927.00 (L. Wang et al., 2024)	0.40 ± 0.00	<0.01
methyl benzoate	100 (Zhang et al., 2020)	0.21 ± 0.02	<0.01

a Odor thresholds were calculated in 45% ethanol/water detected in this study.

In this study, sotolon was confirmed to contribute to herb and smoky aromas with OAV = 56. It not only has unique aroma characteristics but also is an important aroma component in alcoholic beverages. For example: in *Baijiu*, sotolon was confirmed to contribute to the aging aroma (FD = 512, OAV = 5) with caramel and herbal aroma (Wang et al., 2023) and the empty cup aroma (FD = 101, OAV = 5) with seasoning-like and herbal aroma (Qin et al., 2024). In *Huangjiu*, sotolon was confirmed as a key odorant of caramel-like aroma (FD = 16–1024, OAV = 1–59) (Chen, Wang, & Xu, 2013; Wang et al., 2022). Thus, more attention needs to be paid to sotolon to explore its role in flavored distilled spirits*.* Anisaldehyde (anise odor, OAV = 89), and acetoin (3-hydroxy-2-butanone, creamy and buttery note, OAV = 51) were found as the important aroma of *Wujiapi* medicinal liquor (Ma et al., 2020; Ma et al., 2022). Isophorone was detected in *Caoyuanwang Baijiu* (woody note, FD = 3) (Wang et al., 2021) and *Feng*-flavor *Chinese Baijiu* as an aged marker (Jia et al., 2022).

### 3.4 Recombination tests

To validate the aroma contributions in medicinal character, we conducted two group reconstitution experiments in the dearomatized *Zhuyeqing* and compared them against the typical original *Zhuyeqing* (*Zhuyeqing-*J0) by trained panelists. The group 1 (dearomatized *Zhuyeqing*) contains 22 components with OAV > 1, which were drawn from our previous research with the same sample (Wang, Han, et al., 2025). Those data were used herein as the comparative data for this experiment (Table S5); the group 2 (adding medicinal compounds in dearomatized *Zhuyeqing*) contains 28 compounds with OAV > 1 (compared the group 1, 6 new identified medicinal compounds with OAV > 1 in F10 (Table 4) were added). Fig. 1B showed that the addition of these substances greatly improved the intensity of the medicinal aroma attributes and the flavor profiles were similar in trend. This means that certain odorants in F10 played a critical role in the medicinal aroma character. Thus, we successfully simulated the typical flavor of *Zhuyeqing*, even though there were still minor differences from the real *Zhuyeqing* samples.

### 3.5 Omission tests

To investigate the potential contributions of these odorants, 11 omitted models were created (Table 5). The differences between each of the omitted models and the full model were compared by a triangle test. The results revealed that M2 (all phenols) and M3 (sotolon) were important groups for *Zhuyeqing*. All of the assessors simply pointed out the omission when missing them, revealing their very highly significant impact on the general aroma. The omission of M4 (all aldehydes and ketones) was evaluated with a highly significant difference.

**Table 5:** Omission tests from the complete aroma reconstitution in the dearomatized *Zhuyeqing* model.

No.	Omitted compounds	n/10	Significance^a^
M1	isophorone	6/10	ns
M2	all phenols	10/10	***
M2–1	eugenol	10/10	***
M2–2	guaiacol	8/10	**
M2–3	*trans*-isoeugenol	10/10	***
M2–4	phenol	5/10	ns
M3	sotolon	10/10	***
M4	all aldehydes and ketones	8/10	**
M4–1	anisaldehyde	7/10	**
M4–2	acetoin	7/10	*
M5	all alcohols	3/10	ns

a “*”, “**”, and “***” indicate significance at p < 0.05, 0.01, and 0.001, respectively.

M2–1 (eugenol) and M2–3(*trans*-isoeugenol), with clove, sweet, and spices characters, were the most important phenols that had a very highly significant impact on the general aroma and medicinal note. M2–2 (guaiacol) and M4–1 (anisaldehyde) with medicinal notes had a highly significant impact on the medicinal aroma. M4–2 (acetoin) was determined with a significant difference. However, when M1 (isophorone), M2–4 (phenol), and M5 (all alcohols) were omitted, there were no significant distinctions. Indicated that these compounds did not significantly contribute to the aroma or influence the general profile through interactions with other odors.

To clarify the contribution of certain odorants on the medicinal aroma characteristic, 10 panelists were asked to score the intensity of this attribute. M2–1 (eugenol), M2–2 (guaiacol), M2–3 (*trans*-isoeugenol), M3 (sotolon), M4–1 (anisaldehyde), and M4–2 (acetoin) omission models were prepared by omitting one of them from the full reconstitution. These compounds were chosen because they were detected in the medicinal aroma fraction, and omission tests had demonstrated their importance. Compared with the complete reconstitution, the sample without eugenol, *trans*-isoeugenol, and sotolon was evaluated with a very highly significant difference in the intensity of medical aroma (seen in Fig. 1C). Besides, the medical aroma intensity of the sample without guaiacol, anisaldehyde, and acetoin has a significant difference from the complete reconstitution. This indicates that eugenol, *trans*-isoeugenol, sotolon, guaiacol, anisaldehyde, and acetoin compounds were important to the medical aroma of *Zhuyeqing*. Thus, we suggested that the foundation of the medicinal aroma profile may be formed by the phenolic compounds (eugenol, isoeugenol, guaiacol) and aldehyde compounds (anisaldehyde, acetoin). Whilst adding the sotolon introduces a crucial nuance of “herbal-smoky” that defines its distinctive character. Moreover, other types of substances are not entirely insignificant either, but rather influence the overall aroma profile of *Zhuyeqing* through the interactions of odorants.

### 3.6 Quantification analysis of medicinal aromatic compounds in bottled vintage *Zhuyeqing*

The concentrations of these 6 medicinal aromas (eugenol, guaiacol, *trans*-isoeugenol, sotolon, anisaldehyde, and acetoin) were quantified in bottled vintage *Zhuyeqing* and simulated *Zhuyeqing*. Results (seen in Fig. 1E (1–6)) showed that the contents of sotolon, eugenol, *trans*-isoeugenol, and guaiacol tended to decrease with aging time. However, the concentrations of anisaldehyde and acetoin were increased with aging time.

In previous studies, the concentration of sotolon was increased during aging and has been identified as an age marker in various alcoholic beverages, including *Baijiu* (L. Wang, Wang, et al., 2024; Wang et al., 2023) *Huangjiu* (Chen, Wang, & Xu, 2013; Zhang et al., 2024), *wine* (Lavigne et al., 2008), and *sake* (Takahashi et al., 1976). However, our research revealed the opposite phenomenon, which may be explained by pH-dependent racemization and distinct degradation pathways of sotolon under different matrix conditions. Pons et al. (2008) noted that the racemization of sotolon occurs through keto-enol tautomerism and was catalyzed by acid in a mildly acidic medium (pH 3–3.5), which might result in either enantiomeric form of sotolon being formed. Dagan et al. (2006) found that the furanone ring of sotolon could be opened in an acid-mediated reaction, resulting in the 1,4-addition of a water molecule onto the corresponding *α*,*β*-unsaturated system and cyclization at pH 1. This mechanism, which occurred in such harsher conditions, may not be suitable for the decomposition of sotolon in natural liquor, but cannot be completely ruled out. Notably, keto-enol tautomerization of furaneol (a related furanone) is pH-dependent (Koga et al., 1998), and furaneol shows optimal stability at pH 3.5 (Roscher et al., 1997). Our measurements of the *Zhuyeqing* sample indicated a pH of 4.1–4.5 (raw data not shown), which differs from the pH 3.0–3.5 reported for wine (Pons et al., 2008), which may partly explain the opposite phenomenon in our study. Currently, research on sotolon degradation mechanisms remains limited and represents a potential direction for future research.

Among the three very important medicinal aromas (sotolon, eugenol, and *trans*-isoeugenol), only sotolon is a chiral isomer. Wang, Wang*,* et al. *(*2024*)* reported that the aroma characteristics of sotolon isomers were distinct, and humans can detect and distinguish these sensory differences. They pointed out that this is due to the biological mechanism of OR8D1, which activates human olfaction. Pons et al. (2008) reported that the distribution of (*R*) and (*S*) forms of sotolon determines its aromatic effect on dry white *wines*. Caillé et al. (2017) found that adding sotolon imparts new aroma notes to the overall sensation of *Chardonnay wine*. Furthermore, various concentration ranges could produce different aroma notes. The variations in aroma of sotolon across different alcoholic beverages may stem from the differing concentrations and presence of sotolon isomers. However, up to now, we still have not confirmed the existing form of sotolon because its isomers cannot be separated only using the FFAP column on GC–MS and GC × GC-TOF/MS. Therefore, we referred to the literature separating and preparing the sotolon enantiomers and analyzed their distribution in *Zhuyeqing* using a chiral column (Pons et al., 2008).

### 3.7 Distribution of the sotolon enantiomers in *Zhuyeqing*

#### 3.7.1 HPLC separation and GC–MS conditions for (*R*)*-*sotolon and (*S*)*-*sotolon

The (*R*)*-*sotolon (peak 1, enantiomeric excess = 99.344%, Fig. S1B) and (*S*)*-*sotolon (peak 2, enantiomeric excess = 99.894%, Fig. S1C) isomers were successfully separated and collected through HPLC analysis (Fig. S1A), as described in the previous method (Wang, Wang, et al., 2024). Each isomer was injected individually to determine its retention times (RTs) on GC–MS via chiral analysis using a *β*-cyclodextrin phase with a polar pre-column. The RTs for the commercial racemic sotolon (Fig. S2C) were as follows: (*R*)*-*sotolon (RT_peak 1_ = 73.5 min, Fig. S2A) and (*S*)*-*sotolon (RT_peak 2_ = 76.5 min, Fig. S2B). The sotolon enantiomers in the *Zhuyeqing-*J0 sample were seen in Fig. S2D.

#### 3.7.2 Perception thresholds and descriptors of the sotolon enantiomers

Enantiomers of chiral odorants can differ in either odor quality, meaning they can elicit different odor sensations, or in odor intensity (Brenna et al., 2003). The aroma characteristics and perception thresholds of the sotolon enantiomers are shown in Table 6. Descriptors are provided for an odor activity value (OAV) of 5. In our research, (*R*)*-*sotolon primarily exhibited sugar, sweet, and plain yogurt aromas, while (*S*)*-*sotolon was mainly associated with smoky, herb, and acid. Both (*R*) and (*S*) forms enantiomers have typical herbal characteristics but evoke different sensations. For instance, (*R*)*-*sotolon resembles the “cool” herb note of the *Mentha haplocalycis* herb, whereas (*S*)*-*sotolon is reminiscent “smoky” herb note of the *Lysimachia foenum-graecum Hance* (*Linglingxiang*) herb. The *Mentha haplocalycis* herb and *Linglingxiang* herb are traditional Chinese herbs that could be used as ingredients for flavored alcoholic beverages. The perception threshold of (*S*)*-*sotolon (0.6 μg/L) in a dilute alcohol solution (45% vol) was over 50 times lower than that of (*R*)*-*sotolon (31.7 μg/L) (see Table 6). These findings confirmed that the stereochemistry of the odorant strongly influences the perception thresholds (Pons et al., 2008).

**Table 6:** Perception thresholds and olfactory descriptors of the sotolon enantiomers in model solution (45% vol of ethanol level).

	perception threshold (μg/L)	descriptors	descriptors in reference
racemic	10.7	smoky, herb	curry, walnut (Pons et al., 2008)
(*R*)*-*sotolon	31.7	sugar, sweet, plain yogurt, *cablin potchouli* herb	walnut, rancid (Pons et al., 2008)
(*S*)*-*sotolon	0.6	smoky, herb, and acid, *Linglingxiang* herb	curry, walnut (Pons et al., 2008)

#### 3.7.3 Distribution of the sotolon enantiomers in *Zhuyeqing* samples

Based on our findings, the concentration distribution of sotolon in bottled vintage tends to decrease. However, the distribution of (*R*) and (*S*) forms sotolon, along with the potential for some interconversion and racemization of chiral compounds, remains uncertain. The enantiomeric distributions of sotolon in *Zhuyeqing* are presented in Table 7. There was a clear distribution pattern with an excess of (*S*) form sotolon, where the ratios (%) of the (*R*) and (*S*) forms sotolon were below 15% and over 85%, respectively. Besides, similar to findings by Pons et al. regarding dry white *wine*, the vintage did not significantly impact the ratio between the (*R*) and (*S*) forms of sotolon (Pons et al., 2008). The concentrations of (*R*)-sotolon were just a little higher than its odor threshold (31.7 μg/L) in *Zhuyeqing-*J0 to *Zhuyeqing-*J6 (38.63–46.23 μg/L) and lower in *Zhuyeqing-*J11 to *Zhuyeqing-*J25 (4.76–29.42 μg/L). The concentrations of (*S*)-sotolon were much higher than its odor threshold (0.6 μg/L) in *Zhuyeqing-*J0 to *Zhuyeqing-*J14 (184.72–358.95 μg/L) and lower in *Zhuyeqing-*J25 (27.89 μg/L). These results demonstrated that (*S*)-sotolon was more important than (*R*)-sotolon in the medicinal aromatic characteristic of *Zhuyeqing.* Besides, both the (*R*) and (*S*) forms sotolon concentrations tended to decrease in the bottled vintage.

**Table 7:** Enantiomer distributions of sotolon in *Zhuyeqing* samples.

	(*R*)*-*sotolon (%)	(*S*)-sotolon (%)	(*R*)*-*sotolon (μg/L)	(*S*)-sotolon (μg/L)
*Zhuyeqing-*J0	11.41 ± 0.81	88.59 ± 0.81	46.23 ± 0.56	358.95 ± 0.60
*Zhuyeqing*-J3	13.37 ± 1.36	86.64 ± 1.36	49.48 ± 1.56	320.65 ± 1.23
*Zhuyeqing*-J6	12.91 ± 1.41	87.09 ± 1.41	38.63 ± 1.25	260.58 ± 1.15
*Zhuyeqing*-J11	10.96 ± 1.54	89.04 ± 1.54	29.42 ± 1.02	238.99 ± 1.35
*Zhuyeqing*-J14	10.51 ± 0.70	89.49 ± 0.77	21.69 ± 0.35	184.72 ± 0.54
*Zhuyeqing*-J25	14.57 ± 1.24	85.43 ± 1.24	4.756 ± 0.23	27.89 ± 0.15

To verify the importance of sotolon, we added real concentrations of the commercial racemic sotolon (sample B), (*R*) and (*S*) forms of sotolon (sample C), to the reconstitution model in the dearomatized *Zhuyeqing* and compared their intensity of medicinal aroma with that of *Zhuyeqing-*J0 (sample A). The result (Fig. 1D) showed that the medicinal aroma intensity of sample C was closer to that of sample A than that of sample B. Therefore, we confirmed that the medicinal aroma of sotolon in *Zhuyeqing* is not caused by a single structure, but is composed of a certain proportion. That may explain why sotolon has different aroma descriptions in various samples. Combining the results of the recombination and omission tests, the high concentration of sotolon (with a high proportion of smoky (*S*)-sotolon) alongside eugenol and *trans*-isoeugenol (sweet, clove), guaiacol (smoky), anisaldehyde (anise), and acetoin compounds creates the unique synergistic medicinal character that defines *Zhuyeqing*.

Above all, considering differences in threshold levels and aroma descriptions, controlling the ratios of the (*R*) and (*S*) forms of sotolon could be an effective method for modulating product flavor.

## 4 Conclusion

Investigating the medicinal characteristics of *Zhuyeqing* makes it easier to directly regulate and develop the product with medicinal aroma, whether enhanced or attenuated. The unique medicinal aroma molecules of *Zhuyeqing* were analyzed using the NPLC-GC × GC-TOF MS method and sensomics approach. In the typical medicinal aroma fraction, 25 odorants were identified, of which 10 odorants (sotolon, eugenol, anisaldehyde, acetoin, (*R, R*)-2,3-butanediol, (*S, S*)-2,3-butanediol, guaiacol, isophorone, phenol, and *trans*-isoeugenol) were found to have OAVs ≥1. Notably, eugenol, sotolon, *trans*-isoeugenol, guaiacol, anisaldehyde, and acetoin were confirmed as the key medicinal aroma compounds positively correlated with the intensity of the medicinal aroma. Furthermore, using a chiral column, we confirmed that the chiral distribution of sotolon in *Zhuyeqing* is predominantly (*S*)-sotolon, which constitutes more than 85% of the composition, while (*R*)-sotolon constitutes less than 15%.

This research highlights the need for further investigation into two key areas in the future: firstly, the degradation mechanism of sotolon, and secondly, the influence of matrix effects on aroma compounds in *Zhuyeqing*. Furthermore, its practical application in the context of production should not be overlooked. The findings of this study serve as a guide for product development and quality, specifically in optimizing the balanced formulation of odor-active compounds and guiding formulation adjustments. By establishing analytical thresholds for process control and integrating these with sensory benchmarks into the product development process, it facilitates the translation of specific research findings into actionable process control parameters. Thus, building a bridge between academic research and practical manufacturing showcases the real-world value of these insights.

## CRediT authorship contribution statement

**Lihua Wang:** Writing – review & editing, Writing – original draft, Visualization, Validation, Methodology, Formal analysis, Data curation, Conceptualization. **Yue Ma:** Writing – review & editing, Writing – original draft, Validation, Supervision, Methodology, Conceptualization. **Ying Han:** Project administration. **Xing Zhang:** Project administration. **Xiaojuan Gao:** Resources. **Wenshuo Li:** Data curation. **Yanhong Bai:** Resources. **Fengxian Wang:** Resources. **Yan Xu:** Supervision, Resources, Project administration, Funding acquisition. **Qun Wu:** Supervision, Resources, Project administration, Investigation. **Ke Tang:** Supervision, Resources, Project administration, Investigation.

## Declaration of competing interest

The authors declare that they have no known competing financial interests or personal relationships that could have appeared to influence the work reported in this paper.

## Data Availability

Data will be made available on request.

## Appendix A Supplementary data

Supplementary material : The progress of the NPLC method (Method S1); the parameters setting for GC–MS/O (Method S2); mixed ISs in quantitative analysis with LLME-GC–MS (Method S3); the progress of making the dearomatized *Zhuyeqing* (Method S4). (A) Separation of a commercial racemic sotolon by high-performance liquid chromatography (HPLC), (B) HPLC analysis of the collected fraction of (*R*)-sotolon (peak 1) and (C) HPLC analysis of the collected fraction of *(S)-*sotolon (peak 2) (Fig. S1); GC–MS (*m*/*z* 128, 83, 55) analysis of (A) the collected fraction of *(R)-*sotolon (peak 1), (B) the collected fraction of *(S)-*sotolon (peak 2), (C) a commercial racemic sotolon, (D) *Zhuyeqing-*J0 organic extract on a β-cyclodextrin column with a 2 m polar precolumn. (Fig. S2). *Zhuyeqing* samples information (Table S1); information of analytical standards (Table S2); definitions and references of aroma attributes (Table S3); validation data for the chemical standards, quantitative ions of odorants using LLE in F10 with the medicinal character of *Zhuyeqing* (Table S4); Concentrations and OAVs of aroma compounds (OAV ≥ 1) in typical *Zhuyeqing-*J0 (as detailed in Table 3 in our earlier paper (Wang et al., 2025a)) (Table S5). Supplementary material
