# Supplementary material for: Decoding the medicinal aromatic characteristic of Zhuyeqing and gaining new insight into sotolon
Source: Food Chem X. 2026 Feb 3;34:103630. doi: 10.1016/j.fochx.2026.103630 (PMC12914837; doi:10.1016/j.fochx.2026.103630)
Supplement: Supplementary material — : The progress of the NPLC method (Method S1); the parameters setting for GC–MS/O (Method S2); mixed ISs in quantitative analysis with LLME-GC–MS (Method S3); the progress of making the dearomatized Zhuyeqing (Method S4). (A) Separation of a commercial racemic sotolon by high-performance liquid chromatography (HPLC), (B) HPLC analysis of the collected fraction of (R)-sotolon (peak 1) and (C) HPLC analysis of the collected fraction of (S)-sotolon (peak 2) (Fig. S1); GC–MS (m/z 128, 83, 55) analysis of (A) the collected fraction of (R)-sotolon (peak 1), (B) the collected fraction of (S)-sotolon (peak 2), (C) a commercial racemic sotolon, (D) Zhuyeqing-J0 organic extract on a β-cyclodextrin column with a 2 m polar precolumn. (Fig. S2). Zhuyeqing samples information (Table S1); information of analytical standards (Table S2); definitions and references of aroma attributes (Table S3); validation data for the chemical standards, quantitative ions of odorants using LLE in F10 with the medicinal character of Zhuyeqing (Table S4); Concentrations and OAVs of aroma compounds (OAV ≥ 1) in typical Zhuyeqing-J0 (as detailed in Table 3 in our earlier paper (Wang et al., 2025a)) (Table S5). [file mmc1.docx]

**Supporting Information**

**Decoding the medicinal aromatic characteristic of *Zhuyeqing* and gaining new insight into sotolon**

**Author's names:**

Lihua Wang ^a, b^, Yue Ma ^a^, Ying Han ^b^, Xing Zhang ^b^, Xiaojuan Gao ^b^, Wenshuo Li ^b^, Yanhong Bai ^b^, Fengxian Wang ^b^, Yan Xu ^a^, Qun Wu ^a^, Ke Tang ^a, *^

^a^ Laboratory of Brewing Microbiology and Applied Enzymology, School of Biotechnology and Key Laboratory of Industrial Biotechnology of Ministry of Education, Jiangnan University, Wuxi 214122, China.

^b^ Laboratory of Analytical, Quality Inspection Center, Key Laboratory of Plant Extraction and Health of Chinese Lujiu (Shanxi), Shanxi Xinghuacun Fenjiu Distillery Co., Ltd., Fenyang, 032205, Shanxi, China.

*To whom correspondence should be addressed. (Tel: +86 510 85918197;

Fax: +86 510 85918201; Ke Tang E-mail: [tandy81@jiangnan.edu.cn](mailto:tandy81@jiangnan.edu.cn))

**Method S1.** The progress of the NPLC method

Firstly, the silica column was washed with methanol, diethyl ether, and pentane of 50mL, respectively. After, the concentrated sample (2 mL) was loaded on the silica column. Finally, ten different eluent solvents (each with 100 mL) with different volume ratios were respectively carried out to separate aroma components at a flow rate of approximately 1 mL/min.

**Method S2.** The parameters setting for GC-MS/O

The programs of oven temperature setting were as following: start at 45 ℃ and hold for 2 min, with a 4 ℃/min ratio to increase to 80 ℃, keep for 1 min, then with a 5 ℃/min ratio to increase to 150 ℃, keep 2 min, finally, with a 10 ℃/min ratio to increase to 230 ℃, hold for 10 min at 230 ℃ (DB-FFAP) or start at 45 ℃ and keep for 2 min, with a 5 ℃/min ratio to increase to 150 ℃, keep for 3 min, then with a 10 ℃/min ratio to increase to 320 ℃, keep for 10 min at 320 ℃ (DB-5). The carrier gas for the column was helium (purity > 99.999%) at a flow rate of 1.5 mL/min. Setting the quadrupole ionization energy at 70 eV with electron ionization mode, the temperature of the ion source was at 250 °C, and the range of mass scan was from m/z 40 to 350.

Osme technology was used to conduct the sensory evaluation by GC-O, where the extracted sample was analyzed directly without dilution. The intensity values were scored, “0-2” indicated “none” to “low”, “2-4” indicated “medium”, and “4-6” indicated high, respectively. The intensity values were averages evaluated by 3 panelists.

**Method S3.** Mixed ISs in quantitative analysis with LLME-GC-MS

The mixed ISs containing 1-butanol-d10 (IS1, 500.00 mg/L), ethyl octanoate-d15 (IS2, 500.00 mg/L), (±)-linalool-d3 (IS3, 100.00 mg/L), l-menthol (IS4, 100.00 mg/L), 2-ethylbutyric acid (IS5, 300.00 mg/L), 2-methoxyphenol-d3 (IS6, 100.00 mg/L), benzyl alcohol-d7 (IS7, 100.00 mg/L) with final concentrations.

**Method S4.** The progress of making the dearomatized *Zhuyeqing*

Dearomatized *Zhuyeqing* was prepared by evaporating it using a Rotavapor (RV 10 digital V Rotary Evaporators, IKA, Germany) with a bath temperature of 20 °C to two-thirds of its initial volume. The evaporated sample was then reconstituted with ethanol and micro-filtered water to obtain the volume and alcohol percentage of the raw *Zhuyeqing*. The dearomatized *Zhuyeqing* was further treated with 5 g/L of resin (LiChrolut EN, 40-120 μm) and continuously stirred for 12 h. The headspace-gas chromatography-mass spectrometry (HS-GC-MS) test confirmed that the resulting dearomatized *Zhuyeqing* did not have any trace compounds involved in this study. The sensory test ensured that dearomatized *Zhuyeqing* had a very low-intensity neutral aroma that could be barely perceived.


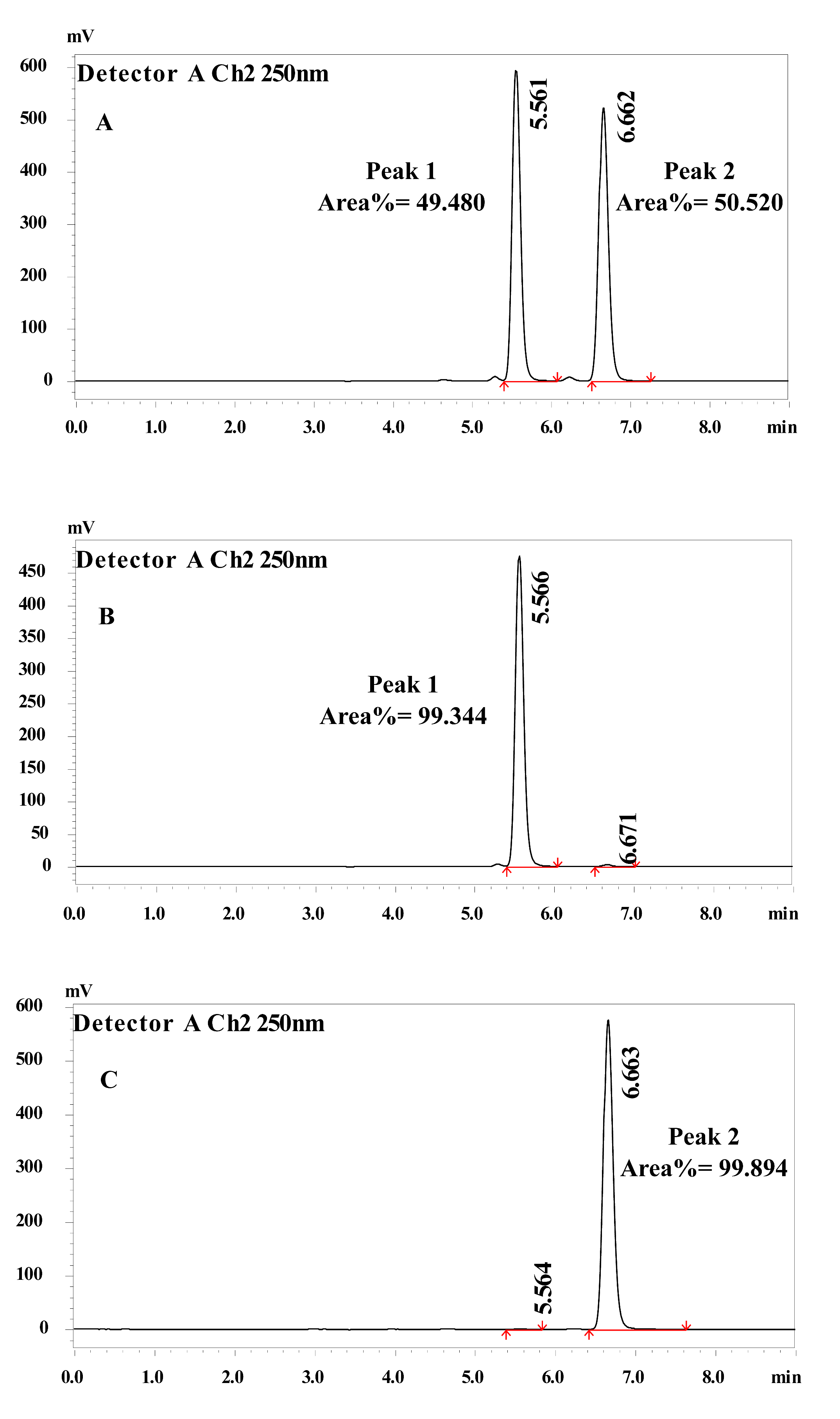


**Figure S1.** (A) Separation of a commercial racemic sotolon by high-performance liquid chromatography (HPLC); (B) HPLC analysis of the collected fraction of (*R*)-sotolon (peak 1); (C) HPLC analysis of the collected fraction of *(S)-*sotolon (peak 2).

**
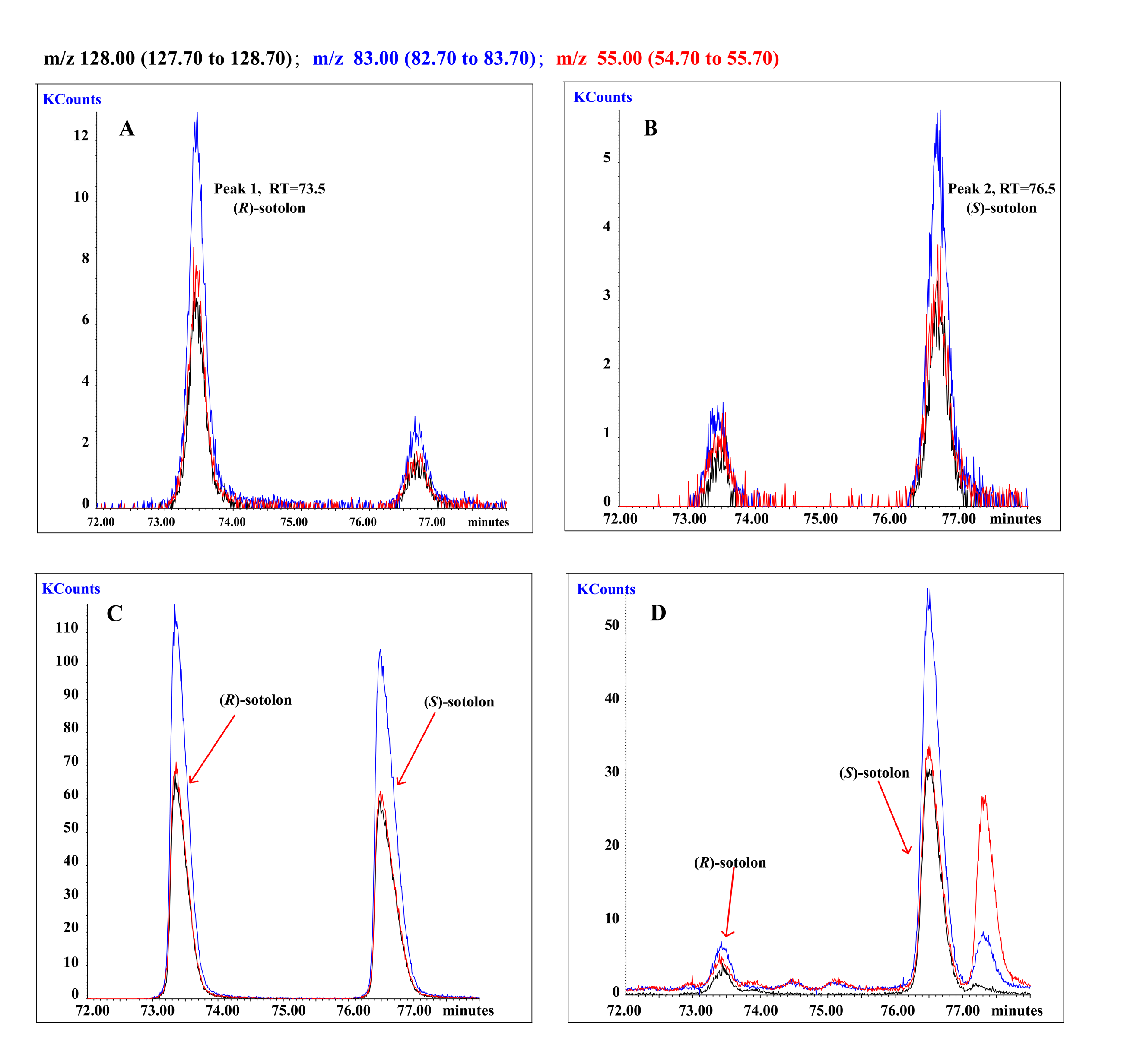
**

**Figure S2.** GC-MS (m/z 128, 83, 55) analysis of (A) the collected fraction of *(R)-*sotolon (peak 1), (B) the collected fraction of *(S)-*sotolon (peak 2), (C) a commercial racemic sotolon, (D) *Zhuyeqing-*J0 organic extract on a β-cyclodextrin column with a 2 m polar precolumn.

**Table S1.** *Zhuyeqing* samples information

| **Typle** | **No.** | **Sample name ^a^** | **Alcohol (% *v/v*)** | **Aging year ^b^** | **Production year** |
| --- | --- | --- | --- | --- | --- |
| vintage | 1 | *Zhuyeqing*-J0 | 45 | 0 | 2024 |
|  | 2 | *Zhuyeqing*-J3 | 45 | 3 | 2021 |
|  | 3 | *Zhuyeqing*-J6 | 45 | 6 | 2018 |
|  | 4 | *Zhuyeqing*-J11 | 45 | 11 | 2013 |
|  | 5 | *Zhuyeqing*-J14 | 45 | 14 | 2010 |
|  | 6 | *Zhuyeqing*-J25 | 45 | 25 | 1999 |

***^a^*** Abbreviation of sample name.

*^b^* The aging year of samples.

**Table S2.** Information of analytical standards

| **No.** | **CAS** | **Name** | **Purity** | **Purchased from** |
| --- | --- | --- | --- | --- |
| 1 | 28664-35-9 | sotolon | ≥98% | ^1^ Innochem |
| 2 | 97-53-0 | eugenol | ≥98% | ^1^ Innochem |
| 3 | 123-11-5 | anisaldehyde | ≥98% | ^1^ Innochem |
| 4 | 513-86-0 | acetoin | ≥99% | ^1^ Innochem |
| 5 | 24347-58-8 | (*R,R*)-2,3-butanediol | ≥98% | ^1^ Innochem |
| 6 | 19132-06-0 | (*S,S*)-2,3-butanediol | ≥98% | ^1^ Innochem |
| 7 | 90-05-1 | guaiacol | ≥99% | ^1^ Innochem |
| 8 | 78-59-1 | isophorone | ≥98% | ^2^ Dikma |
| 9 | 108-95-2 | phenol | ≥98% | ^1^ Innochem |
| 10 | 5932-68-3 | *trans*-isoeugenol | ≥99% | ^1^ Innochem |
| 11 | 1193-18-6 | 3-methyl-2-cyclohexen-1-one | ≥99% | ^2^ Dikma |
| 12 | 121-33-5 | vanillin | ≥99% | ^3^ Sigma-Aldrich |
| 13 | 7786-61-0 | 4-vinylguaiacol | ≥98% | ^3^ Sigma-Aldrich |
| 14 | 55418-52-5 | piperonyl acetone | ≥99% | ^3^ Sigma-Aldrich |
| 15 | 100-52-7 | benzaldehyde | ≥97% | ^3^ Sigma-Aldrich |
| 16 | 60-12-8 | 2-phenylethanol | ≥99% | ^3^ Sigma-Aldrich |
| 17 | 91-10-1 | 2,6-dimethoxyphenol | ≥99% | ^3^ Sigma-Aldrich |
| 18 | 471-01-2 | *β*-isophorone | ≥98% | ^3^ Sigma-Aldrich |
| 19 | 98-86-2 | acetophenone | ≥99% | ^3^ Sigma-Aldrich |
| 20 | 104-46-1 | anethole | ≥97% | ^3^ Sigma-Aldrich |
| 21 | 100-51-6 | benzyl alcohol | ≥99% | ^3^ Sigma-Aldrich |
| 22 | 89-81-6 | piperitone | ≥99% | ^3^ Sigma-Aldrich |
| 23 | 93-58-3 | methyl benzoate | ≥99% | ^3^ Sigma-Aldrich |
| IS1 | 1219798-38-5 | ethyl octanoate-d15 | ≥99% | ^5^ Trc |
| IS2 | 34193-38-9 | 1-butanol-d10 | ≥99% | ^4^ Aladdin |
| IS3 | 2216-51-5 | L-menthol | ≥99% | ^4^ Aladdin |
| IS4 | 74495-69-5 | 2-methoxyphenol-d3 | ≥99% | ^6^ Tmrm |
| IS5 | 71258-23-6 | benzyl alcohol-d7 | ≥99% | ^6^ Tmrm |
| IS6 | 156420-66-5 | sotolon-^13^C2 | 100μg/mL | ^7^ Zzbio |

^1^ Innochem: Innochem Science & Technology Co., Ltd. (Beijing, China).

^2^ Dikma: Dikma Technologies Inc. (Beijing, China).

^3^ Sigma-Aldrich: Sigma-Aldrich Co., Ltd. (Shanghai, China).

^4^ Aladdin: Aladdin Biochemical Technology Co., Ltd. (Beijing, China).

^5^ Trc: Toronto Research Chemicals Inc. (Toronto, Canada).

^6^ Tmrm: TMRM Quality Inspection Technology Co., Ltd. (Changzhou, China).

^7^ Zzbio: ZZBIO CO., LTD. (Shanghai, China).

**Table S3.** Definitions and references of aroma attributes**.**

| **No.** | **Aroma** | **Definition** | **Reference (in 45% *v*/*v* ethanol/water)** |
| --- | --- | --- | --- |
| 1 | medicinal | aroma of herbs | ethyl 2-hydroxybutanoate (50 mg/L) |
| 2 | sweet | aroma similar to honey and sweet fruits | *β*-damascenone (250 μg/L*)* |
| 3 | alcoholic | aromas presented by alcohols | 45% *v*/*v* ethanol/water |
| 4 | woody | like wood | extraction of Muxiang |
| 5 | floral | similar to the aroma of florals | extraction of Juhua |
| 6 | fruity | aroma like ripe fruits | ethyl acetate (2 g/L) and ethyl hexanoate (10 mg/L) |
| 7 | smoky | similar to smoke | sotolon (1 mg/L) |
| 8 | acid | aroma presented by volatile acidic components | acetic acid (1 g/L) |
| 9 | nutty | similar to roasted almond seeds | roasted almond seeds |

**Table S4** Validation data for the chemical standards, quantitative ions of odorants using LLE in F10 with the medicinal character of *Zhuyeqing*

| **Compounds** | **Quantitative** | ***IS** | **Quantitative ion (*m*/*z*)** | **Calibration Curve** | | | **Range** | **LOD** | **LOQ** |
| --- | --- | --- | --- | --- | --- | --- | --- | --- | --- |
|  | **method** |  |  | **Slope** | **Intercept** | **R^2^** | **(μg/L)** | **(μg/L)** | **(μg/L)** |
| sotolon | LLE | IS6 | 83 | 0.8855 | 0.3308 | 0.9610 | 60-75000 | 1.41 | 4.70 |
| eugenol | LLME | IS4 | 164 | 0.9889 | 0.4755 | 0.9988 | 5-302340 | 1.00 | 5.00 |
| anisaldehyde | LLE | IS5 | 135 | 0.0269 | -0.0090 | 0.9932 | 14-23400 | 3.57 | 7.15 |
| acetoin | LLE | IS1 | 45 | 0.0582 | 0.0008 | 0.9957 | 94-950000 | 40.00 | 90.00 |
| (*R,R*)-2,3-butanediol | LLE | IS2 | 45 | 1.7228 | -0.0600 | 0.9136 | 10000-350000 | 10.00 | 20.00 |
| (*S,S*)-2,3-butanediol | LLE | IS2 | 45 | 0.1748 | -0.0215 | 0.9930 | 3000-700000 | 7.00 | 14.00 |
| guaiacol | LLME | IS4 | 109 | 1.6209 | -0.0107 | 0.9993 | 30-10000 | 0.50 | 2.04 |
| isophorone | LLE | IS3 | 82 | 2.3590 | -0.0161 | 0.9944 | 300-3000 | 2.54 | 5.08 |
| phenol | LLME | IS5 | 94 | 2.5256 | 0.0045 | 0.9979 | 18.45-1845 | 3.69 | 7.31 |
| *trans*-isoeugenol | LLE | IS4 | 164 | 0.1110 | -0.0210 | 0.9930 | 165-41600 | 3.30 | 33.00 |
| 3-methyl-2-cyclohexen-1-one | LLE | IS3 | 82 | 0.8247 | -0.0034 | 0.9986 | 277-40861 | 138.00 | 270.00 |
| vanillin | LLE | IS4 | 151 | 6.2539 | -0.0601 | 0.9984 | 280-7500 | 2.04 | 6.80 |
| 4-vinylguaiacol | LLE | IS4 | 150 | 0.9611 | -0.0080 | 0.9989 | 15-10000 | 5.28 | 10.36 |
| piperonyl acetone | LLE | IS4 | 135 | 0.3038 | -0.0079 | 0.9930 | 65-4900 | 2.00 | 60.00 |
| benzaldehyde | LLME | IS5 | 106 | 0.9835 | 0.0003 | 0.9984 | 30-7000 | 10.00 | 16.00 |
| 2-phenylethanol | LLME | IS5 | 91 | 2.7553 | 0.4170 | 0.9933 | 360-66000 | 72.00 | 360.00 |
| 2,6-dimethoxyphenol | LLE | IS4 | 154 | 0.0633 | -0.0324 | 0.9718 | 400-4000 | 100.00 | 200.00 |
| *β*-isophorone | LLE | IS3 | 81 | 0.2953 | -0.0372 | 0.9928 | 14.5-3683 | 3.68 | 7.37 |
| acetophenone | LLE | IS5 | 105 | 2.9555 | 0.0339 | 0.9922 | 30-4000 | 0.19 | 0.65 |
| anethole | LLE | IS2 | 148 | 0.2276 | 0.0006 | 0.9992 | 10-5000 | 4.59 | 9.17 |
| benzyl alcohol | LLME | IS5 | 108 | 1.1002 | -0.0018 | 0.9950 | 140-7000 | 70.00 | 140.00 |
| piperitone | LLE | IS3 | 82 | 0.4786 | -0.0014 | 0.9952 | 5-300 | 0.09 | 0.30 |
| methyl benzoate | LLE | IS5 | 85 | 4.7892 | 0.0122 | 0.9957 | 6-1200 | 1.72 | 3.43 |
| (*R*)-sotolon | LLE | IS6 | 83 | 16.9460 | -0.6339 | 0.9951 | 200-3000 | 100.00 | 150.00 |
| (*S*)-sotolon | LLE | IS6 | 83 | 15.4140 | -0.3232 | 0.9909 | 200-3000 | 100.00 | 150.00 |

*IS: the internal standard used to quantitate the compounds: ethyl octanoate-d15 (IS1), 1-butanol-d10 (IS2), l-menthol (IS3), 2-methoxyphenol-d3 (IS4), benzyl alcohol-d7 (IS5), sotolon-13C2 (IS6).

**Table S5.** Concentrations and OAVs of aroma compounds (OAV ≥ 1) in typical *Zhuyeqing-*J0 (as detailed in Table 3 in our earlier paper (Wang et al., 2025)).

| **No.** | **Compounds** | **Concentrations (μg/L)** | **OAV** |
| --- | --- | --- | --- |
| 1 | ethyl cinnamate | 290.06 ± 16.88 | 414.37 |
| 2 | β-damascenone | 16.65 ± 1.35 | 166.50 |
| 3 | ethyl octanoate | 1113.92 ± 120.50 | 86.35 |
| 4 | ethyl hexanoate | 3107.04 ± 234.61 | 56.15 |
| 5 | ethyl acetate | 1696667.43 ± 230056.14 | 52.05 |
| 6 | d-limonene | 1302.01 ± 42.04 | 38.29 |
| 7 | ethyl butanoate | 2596.35 ± 268.14 | 31.86 |
| 8 | eugenol | 13617.04 ± 388.13 | 28.97 |
| 9 | 3-methylbutyl acetate | 1305.53 ± 58.25 | 13.90 |
| 10 | β-myrcene | 40.32 ± 3.15 | 8.23 |
| 11 | ethyl pentanoate | 144.23 ± 8.13 | 5.38 |
| 12 | phenylacetaldehyde | 114.59 ± 9.99 | 4.58 |
| 13 | 2-methyl-1-propanol | 4666.62 ± 24.52 | 4.46 |
| 14 | 3-methylbutanoic acid | 114253.65 ± 5675.61 | 4.04 |
| 15 | bornyl acetate | 210.51 ± 5.47 | 2.81 |
| 16 | guaiacol | 23.70 ± 0.39 | 2.50 |
| 17 | 1-nonanol | 125.23 ± 20.3 | 2.50 |
| 18 | 3-methyl-1-butanol | 396642.77 ± 25104.53 | 2.22 |
| 19 | phenol | 59.43 ± 4.05 | 1.98 |
| 20 | ethyl 2-hydroxybutanoate | 1441.25 ± 82.99 | 1.80 |
| 21 | *trans-*isoeugenol | 35.53 ± 1.62 | 1.62 |
| 22 | ethyl 3-phenylpropanoate | 192.95 ± 37.03 | 1.54 |

**REFERENCE**

Wang, L., Han, Y., Zhang, X., Gao, X., Xu, Y., Wu, Q., & Tang, K. (2025). Characterization of key aroma compounds of Zhuyeqing by aroma extract dilution analysis, quantitative measurements, aroma recombination, and omission studies. *Foods, 14*(3). <https://doi.org/10.3390/foods14030344>.
